# Microbial pectinases: an ecofriendly tool of nature for industries

**DOI:** 10.1007/s13205-016-0371-4

**Published:** 2016-02-08

**Authors:** G. Garg, A. Singh, A. Kaur, R. Singh, J. Kaur, R. Mahajan

**Affiliations:** 1 Department of Biotechnology, Maharishi Markendeshwar University, Mullana, Ambala, India; 2 Department of Biotechnology, Kurukshetra University, Kurukshetra, India; 3 Department of Biotechnology, Panjab University, Chandigarh, India

**Keywords:** Clarification, Bioscouring, Recycling, Degumming, Biobleaching

## Abstract

Pectinases are the growing enzymes of biotechnological sector, showing gradual increase in their market. They hold a leading position among the commercially produced industrial enzymes. These enzymes are ecofriendly tool of nature that are being used extensively in various industries like wine industry; food industry; paper industry for bleaching of pulp and waste paper recycling; in the processing of fruit–vegetables, tea–coffee, animal feed; extraction of vegetable oil and scouring of plant fibres. Moreover, enzymatic catalysis is preferred over other chemical methods, since it is more specific, less aggressive and saves energy. This is the review which covers the information available on the applicability potential of this group of enzymes in various sectors.

## Introduction

**Fig. 1:**
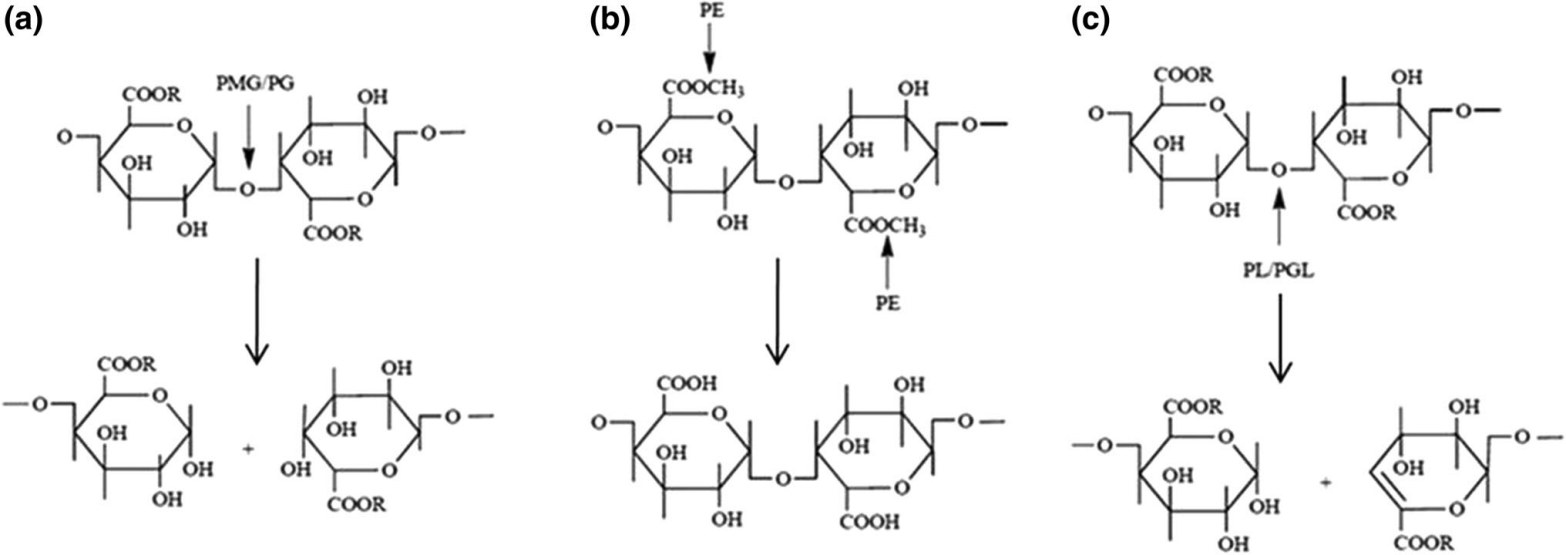
Mode of action and products of pectinases (Lang and Dornenburg 2000). Mode of action of pectinases: **a** R=H for PG (Polygalacturonases) and CH_3_ for PMG (Polymethylgalacturonases), **b** PE (Pectin esterase), **c** R=H for PGL (Polygalacturonate lyase) and CH_3_ for PL (Pectin lyase). The *arrow* indicates the mode of action of different forms of pectinases. Products of pectinases: **a** Saturated galacturonic acid formed by PG and Saturated methoxylated galacturonide by PMG, **b** Pectic acid formed by PE, **c** Unsaturated galacturonic acid formed by PGL and unsaturated methoxylated galacturonide by PL

The primary source of industrial enzymes is microorganisms, out of which, 50 % originate from fungi and yeast, 35 % from bacteria, while the remaining 15 % are either of plant or animal origin (Anisa and Girish 2014). The pectinases are being produced by various kinds of microorganisms (Servili et al. 1992; Kapoor et al. 2001; Angayarkanni et al. 2002; Hoondal et al. 2002; Sharma and Satyanarayana 2012; Sharma et al. 2013b; Mohamadi et al. 2014). They are also reported to be produced in combination with other industrially important enzymes by the same microbial isolate (Kaur et al. 2011; Singh et al. 2015). The pectinase enzyme is broadly classified into three types on the basis of their mode of action: pectin esterase, hydrolases and lyases. Pectin esterase catalyses the de-esterification of the methoxyl group of pectin, forming pectic acid. Hydrolases (Polygalacturonases and Polymethylgalacturonases)—Catalyses the hydrolytic cleavage of α-1,4-glycosidic linkage in pectic acid and pectin, respectively, while Lyases (Polygalacturonate Lyase and Polymethylgalacturonate Lyase)—Catalyses the cleavage of α-1,4-glycosidic linkage in pectic acid and pectin, respectively by trans-elimination reaction and forming unsaturated galacturonates and methyl galacturonates, respectively. The Classification of Pectinases, their mode of action and products formed are shown in Table 1 and Fig. 1, respectively. Pectinases can be produced by both submerged and solid state fermentation (SSF). Optimised conditions for pectinase production by various microorganisms have been shown in Table 2.

**Table 1:** Classification of pectinases (data modified from Jayani et al. 2005)

E.C. suggested name	Common name	E.C. No.	Substrate	Mode of action and cleavage	Product
*De-esterifying enzymes*					
Polymethylgalacturonate esterase (PMGE)	Pectin esterase	3.1.1.11	Pectin	Random cleavage of methyl ester group of galacturonate unit	Pectic acid + methanol
*De-polymerising enzymes*					
(a) Hydrolases					
(i) Polygalacturonases (PG)—Catalyzes the hydrolytic cleavage of α-1,4-glycosidic linkage in pectic acid					
Endopolygalacturonase (endo—PG)	Polygalacturonase	3.2.1.15	Pectate	Random cleavage of pectic acid	Oligo-galacturonates
Exopolygalacturonase 1 (exo—PG1)	Polygalacturonase	3.2.1.67	Pectate	Terminal cleavage from the non reducing end of the polygalacturonic acid	Mono-galacturonates
Exopolygalacturonase 2 (exo—PG2)	Polygalacturonase	3.2.1.82	Pectate	Penultimate Cleavage	Di-galacturonates
(ii) Polymethylgalacturonases (PMG)—Catalyses the hydrolytic cleavage of α-1,4-glycosidic linkage in pectin					
Endo—PMG	Pectin hydrolase		Pectin	Random cleavage	Oligo methyl-galacturonates
Exo—PMG	Pectin hydrolase		Pectin	Terminal cleavage from the non-reducing end of pectin	Methyl mono-galacturonate
(b) Lyases					
(i) Polygalacturonate Lyase (PGL)—Catalyses the cleavage of α-1,4-glycosidic linkage in pectic acid by trans-elimination forming unsaturated galacturonates					
Endo—PGL	Pectate lyase	4.2.2.2	Pectate	Random cleavage	Unsaturated oligo-galacturonates
Exo—PGL	Pectate lyase	4.2.2.9	Pectate	Cleavage of penultimate bonds from non-reducing end	Unsaturated di-galacturonates
Oligogalacturonate lyase	Pectate lyase	4.2.2.6	Oligo-galacturonate	Terminal cleavage	Unsaturated mono-galacturonates
(ii) Polymethylgalacturonate Lyase (PMGL)—Catalyses cleavage of α-1,4-glycosidic linkage in pectin by trans-elimination forming unsaturated methyl galacturonates at the non-reducing end					
Endo –PMGL	Pectin lyase	4.2.2.10	Pectin	Random cleavage	Unsaturated methyl oligo-galacturonates
Exo—PMGL	Pectin lyase		Pectin	Terminal cleavage	Unsaturated methyl mono-galacturonates

**Table 2:** Fermentation conditions for pectinase production by various microorganisms

Microorganism	Substrate	Fermentation			References
		Type	Temperature (°C)	pH	
*Aspergillus niger* A 138	Sucrose	SmF	32	4.5	Friedrich et al. (1990)
*Aspergillus niger* 3T5B8	Wheat bran	SSF	32	–	Couri et al. (2000)
*Bacillus *sp. DT7	Pectin	SmF	37	7.2	Kashyap et al. (2000)
*Penicillium veridicatum* RFC3	Orange bagasse, Wheat bran	SSF	30	–	Silva et al., 2002
*Bacillus* sp. *DT7*	Wheat bran	SSF	37	–	Kashyap et al. (2003)
*Aspergillus fumigates*	Wheat bran	SSF	50	–	Phutela et al. (2005)
*Aspergillus niger*	Sunflower head	SSF	30	5.0	Patil and Dayanand (2006)
*Aspergillus fumigatus* MTCC 870	Wheat flour	SmF	30	5.0	Palaniyappan et al. (2009)
*Penicillium chrysogenum*	Sucrose	SmF	35	6.5	Banu et al. (2010)
*Aspergillus heteromorphus*	Orange peel	SmF	30	4.5	Mandhania et al. (2010)
*Thermomucor indicae*-*seudaticae*	Wheat bran, Orange bagasse	SSF	45	–	Martin et al. (2010)
*Penicillium* sp.	Pectin	SSF	35	6.0	Patil and Chaudhari (2010)
*Fomes sclerodermeus*	Soy and Wheat bran	SSF	28	–	Salariato et al. (2010)
*Bacillus subtilis*	Pectin	SmF	50	7.0	Swain and Ray (2010)
*Bacillus* sp. AD 1	Pectin	SmF	37	7.0	Dey et al. (2011)
*Aspergillus niger*	Pectin	SmF	37	5.5	Gomes et al. (2011)
*Aspergillus sojae M3*	Orange peel	SSF	22	–	Demir et al. (2012)
*Aspergillus flavus*	Orange peel	SSF	40	5.5	Johnson et al. (2012)
*Penicillium atrovenetum*	Orange peel	SSF	40	5.0	Johnson et al. (2012)
*Aspergillus oryzae*	Orange peel	SSF	35	5.5	Johnson et al. (2012)
*Bacillus subtilis*	Date syrup	SmF	45	8.0	Qureshi et al. (2012)
*Pseudozyma *sp. SPJ	Citrus peel	SSF	32	7.0	Sharma et al. (2012)
Mixed culture of *Aspergillus fumigatus,* *Aspergillus sydowii*	Pineapple residue	SSF	35	5.0	Singh and Mandal (2012)
*Aspergillus niger*	Sour oranges peel	SSF	30	5.0	Vasanthi and Meenakshisundaram (2012)
*Streptomyces* sp.	Pectin	SmF	30	8.5	Das et al. (2013)
*Penicillium citrinum*	Sugar beet pulp	SSF	30	5.5	EI-Batal et al. (2013)
*Erwinia carotovora*	Pectin	SmF	35	5.2	Kothari and Baig (2013)
*Bacillus firmus*	Pectin	SmF	50	7.0	Roosdiana et al. (2013)
*Aspergillus niger*	Date pomace	SmF	–	6.18	Seifollah and Khodaverdi (2013)
*Rhizomucor pusillus*	Pectin	SSF	45	5.0	Siddiqui et al. (2013)
*Rhodotorula glutinis* MP-10	Citrus pectin	SmF	30	5.5	Taskin (2013)
*Aspergillus sojae*	Wheat bran	SSF	37	6.0	Demir and Tari (2014)
*Aspergillus niger* HFD5A-1	citrus pectin	SmF	30	4.5	Ibrahim et al. (2014)
*Trichoderma viridi*	Orange peel	SSF	30	5.5	Irshad et al. (2014)

## Industrial applications

**Fig. 2:**
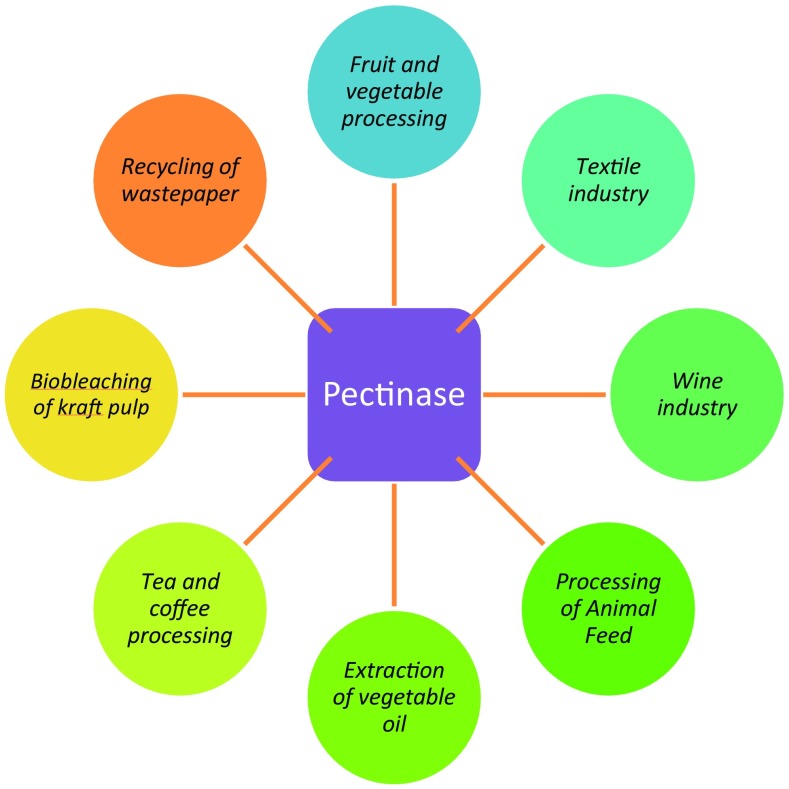
Applications of pectinases in various industries

The first commercial application of pectinases was reported in 1930 by Kertesz for the clarification of apple juice. List of companies producing commercial pectinases is given in Table 3. The application aspect of pectinases has been discussed under the following heads (Fig. 2).

**Table 3:** List of companies producing commercial pectinases (Data modified from Kashyap et al. 2001a)

Product trade name	Manufacturer
Pectinase	Biocon Pvt Ltd, India
Pectolase	Grinsteelvaeket, Denmark
Pectinase Mash	Novozyme, Denmark
Ultrazyme	Ciba-Geigy A.G., Switzerland
Klerzyme	Clarizyme Wallerstein, Co., USA
MaxLiq	Danisco, Denmark
Sclase	Kikkoman Shoyu, Co., Japan
Pectinex	Schweizerische Ferment, A.G., Switzerland
Pectinex Ultra SP-L and Pectinex CLEAR	Novo Nordisk Ferment Ltd., Switzerland
Pectinol	Rohm, GmbH, West Germany
Ly Peclyve PR	Lyven, France
Panzym	C.H. Boehringer Sohn, West Germany
Rohapect MA Plus	AB Enzymes, Finland
Rapidase	Societe Rapidase, S.A., France
Solpect L 60	Varuna Biocell Pvt. Ltd., India
Food Grade Pectinase	Unikbio Biotech Ltd., China

### Fruits and vegetables processing

A general practice in fruit and vegetable processing is the treatment of pulp with appropriate enzyme preparations (Naidu and Panda 1998; Ramadan et al. 2007; Chaudhri and Suneetha 2012; Khan et al. 2013). Juices with low viscosity, high clarity and high in nutrition are more desirable by consumers.

Pectinases play a crucial role to reduce the viscosity, increase the yield and clarification of juice by liquefaction of pulps, remove off the peels (Kashyap et al. 2001a; Kareem and Adebowale 2007; Chaudhri and Suneetha 2012; Makky and Yusoff 2015) and in maceration of vegetables to produce various products like pastes and purées (Sreenath et al. 1994; Demir et al. 2000; Tochi et al. 2009). Depectinization depends upon the composition of juice, the type of enzyme used and the time consumed (Versari et al. 1998). The pH conditions for depectinization of juices (orange, dragon, apple, pear, grapes, guava, banana, papaya, carrot, beet etc.) with pectinase has been varied from pH 2.5–6 (Soares et al. 2001; Croaka and Corredig 2006), treatment time range from 5 min to 6 h (Soares et al. 2001; Singh and Gupta 2004; Tochi et al. 2009; Aliaa et al. 2010), temperature range below 50 °C (Kashyap et al. 2001a; Soares et al. 2001; Singh and Gupta 2004; Aliaa et al. 2010) and enzyme dose range from 0.06 to 0.135 % v/w (Singh and Gupta 2004; Aliaa et al. 2010; Dang et al. 2012).

In citrus juice processing, pectic enzymes contribute in the removal of cloudiness and stabilisation of juice (Braddock 1981). Pectinolytic enzymes have also been applied in association with other cell wall degrading enzymes such as cellulases and hemicellulases (Bhat 2000). Croaka and Corredig (2006) reported the changes occurring to orange juice cloud particles after addition of polygalacturonase and pectin esterase. The addition of polygalacturonase showed no effect on the particle size of juice cloud, while the addition of pectin esterase causes the aggregation of the cloud particles within a few minutes at the natural pH (3.8) of the juice, and the amount of enzyme added, affected the kinetics of the aggregation.

As the banana and papaya are soft fruits and contain high level of soluble pectin, maceration of these fruits resulted in semigelled mass that was very difficult to press (Pilnik and Voragen 1993). Use of pectinase enzyme resulted in pulp with better pressing characteristics and higher juice yield. Crushing and macerating papaya and banana by enzymatic treatment resulted in extraction of 60 to 95 ml juice per 100 g of material, which is about three-to-four fold more as compared to the control. Soares and coworkers (2001) reported that the yield of fruit (pear, guava, banana, papaya) and vegetable (carrot, beet) juice was improved significantly by pectinase treatment and the material was pressed more easily than the control and the residual dry weight of solid residues decreased in the range from 5 to 64 %. The yield obtained from milling carrots treated with enzyme solution from *Bacillus* Ar1.2, Ega16 and Ega22 strains was 40–50 ml juice per 100 g of material, i.e., two-fold higher than the control (20 ml/100 g).

About 25 % increase in pineapple juice recovery was obtained, when a mixture of two commercial enzymes pectinase and hemicellulase was used at the extraction temperature of 40 °C against control (Tochi et al. 2009). The use of pectinase and hemicellulase preparations in pineapple pulp not only increased the juice recovery but also ensured the highest possible quality of the end products (Kilara 1982; Kashyap et al. 2001a). Increased juice yield is mainly due to the ability of enzymes to degrade the cell walls, thus significantly lowers the viscosity of the recovered juices and hence minimises membrane fouling during filtration operations (de Carvalho et al. 2008; Chaudhri and Suneetha 2012).

Pectinase treatment also resulted in about 143 % more transmittance and 35.5 % drop in viscosity as compared to control, in case of apple juice. Enzymatically clarified juices did not show any significant haze development, when stored at room temperature (~25 °C) after 2 months of storage. Singh and Gupta (2004) also reported the effect of gelatin on the efficacy of pectinolytic enzyme from *Aspergillus niger* for clarification of apple juice. Apple juice, when treated with 15 IU/ml of enzyme in presence of 0.01 % gelatin, was about 1.5–2 times more clarified as compared to control containing only enzyme, at 45 °C with holding time of 6 h. Extraction by enzymatic maceration can increase the yield by more than 90 % as compared to conventional mechanical juicing, and also improves the organoleptic, nutritional properties and filtration efficiency (Rombouts et al. 1980). The partially purified pectinase from *Bacillus* VIT sun-2 in combination with commercial cellulase and xylanase has more efficacy in increasing the yield and clarification of apple juice followed by grape, orange and pomegranate juice and its effect increases with increase in incubation time and enzyme concentration (Praveen and Suneetha 2015). Pomace liquefaction may also be used to obtain value-added foods, as it offers the opportunity of releasing apple polyphenols and polysaccharides to a greater extent. When the apple juice is ultrafiltered, the permeate flux of depectinized juice is much higher than pectinized juice. The reduction in apple juice viscosity and total pectin content resulted in increase of permeation rate.

The treatment of fruit with pectinase helps in the release of phenolic content from the fruit skin (Sharma et al. 2013a). These phenolic components play a significant contributory role as an antioxidant and this is important in the maintenance of health and protection from coronary heart disease and certain cancers (Miller and Rice-Evans 1997). Phenolics content was higher up to 15 % in the enzyme treated samples which suggest that dragon fruit beverage is rich in antioxidant capacity than the unprocessed beverage (Aliaa et al. 2010).

Some reports are available on the simultaneous use of ultrasound and pectinase (Lieu and Le 2010; Dang et al. 2012). Yield of juice was more in case of acerola and grape mashes, when treated with ultrasound and pectinase simultaneously as compared to either ultrasonic or enzymatic treatments (Lieu and Le 2010; Dang et al. 2012). The maximum extraction yield in acerola mash, when treated with pectinase at the concentration of 0.12 % v/w for 26.3 min in the presence of ultrasound was 87.4 % which is 3.2 and 15.5 % higher than by the ultrasonic and enzymatic treatment, respectively (Dang et al. 2012). It can be explained that, ultrasound generates collapsing cavitational bubbles, the energy of which provides greater penetration of the solvent into the cellular material and enhances mass transfer to and from interfaces; in addition, acoustic cavitation can disrupt the cell walls and release the cellular materials which in turn cause increase in extraction yield (Patist and Bates 2008).

### Wine processing

The main functions of pectinolytic enzymes in the wine making process are to support the extraction process, maximise juice yield, facilitate filtration and intensify the flavour and colour (Chaudhri and Suneetha 2012). Enzymatically treated wines showed more stability with reduced filtration time in comparison to control wines (Blunt 2000; Jayani et al. 2005). Treatment of macerated fruits with pectinolytic enzymes, before the addition of inoculum resulted in improved characteristics of wine (Revilla and Ganzalez-san 2003; Praveen and Suneetha 2014). Clarification of must prior to the onset of alcoholic fermentation also improves the sensory characteristics of white wine (Reddy and Reddy 2009). Bosso (1993) reported the higher levels of alcohol production in fermented grape must, pre-treated with pectolytic enzymes and observed increase in iso-amyl alcohol and 2-phenyl ethanol and a decrease in n-propanol concentrations. Reddy and Reddy (2009) studied the combined effect of pectinase treatment and fermentation by yeast cultures on ethanol production. Pectinase treatment increased the yield of juice, when treated at 0.6 % of enzyme concentration and fermentation was conducted at 30 °C and pH 4.5 for a period of 12 h. The increase in ethanol may be due to the clarification of mango must and increased sugar concentration in pectinase treated samples.

Various reports have shown that, the addition of pectinolytic enzymes in the wine making process leads to increased levels of methanol in wine due to the activity of pectin esterase (Servili et al. 1992; Revilla and González-SanJosé 1998). Methanol is toxic and its maximum concentration in wine should be regulated. Therefore, pectin esterase should be at low concentrations in commercial mixtures.

### Saccharification of agricultural substrates

Pectinases are also being used in biorefineries for hydrolyzing pectin present in pectin-rich agro-industrial wastes (Biz et al. 2014). These wastes are processed into simple sugars so that they could be converted into bioethanol or used as fermentable sugars (Collares et al. 2012; Hossain et al. 2011). Different enzymes such as pectinase, hemicellulases and cellulases are being used to convert polysaccharides present in the plant cell wall into simple sugars (Beldman et al. 1984). Treatment of *Landoltia punctata* (duckweed) with a pectinase dose of 26.54 pectin transeliminase unit/g mash at 45 °C for 5 h resulted in about 142 % increase in glucose as compared to the untreated mash. This glucose is further used in the production of 30.8 ± 0.8 g/L ethanol concentration using duckweed as the feedstock (Chen et al. 2012).

### Extraction of vegetable oil

Vegetable oils of olive, sunflower, coconut, palm or canola are obtained by extraction with organic solvents such as hexane, which is a potential carcinogen (Kashyap et al. 2001a). The use of pectolytic enzymes, in this case preferably alkaline, allows the extraction of vegetable oils in an aqueous process by degradation of cell wall components. Now days, the use of enzyme preparations containing cellulases, hemicellulases and pectinases has begun for maximum extraction of oil. Enzymatic treatment resulted not only in increase of oil yield but also increased the polyphenolic and vitamin E content, thereby also enhanced its organoleptic quality (Kashyap et al. 2001a; Hoondal et al. 2002; Iconomou et al. 2010).

### Processing of textile material

The conventional scouring process involves the use of harsh chemicals and is slowly being replaced with the ecofriendly approach by using enzymes. Bio-scouring is an ecofriendly method for removal of non-cellulosic impurities from the fiber with specific enzymes (Praveen and Suneetha 2014). It makes the fibre surface more hydrophilic (Li and Hardin 1998). Bioscouring also avoids high energy consumption and severe pollution problems that are associated with conventional alkaline scouring (Rajendran et al. 2011). Pectinases also prevent fiber damage (Jayani et al. 2005; Klug-Santner et al. 2006). Alkaline pectinase has been considered as the most suitable enzyme for cotton scouring by many researchers, because the degradation and elimination of pectin facilitates the removal of loosened waxes (Tzanov et al. 2001; Wang et al. 2007). Whereas some scientists have also studied the effect of acidic and neutral pectinase on the cotton bioscouring (Pusic et al. 2015). Improved results were achieved, when pectinase have been used in conjunction with amylases, lipases, cellulases and hemicellulases to remove sizing agents from cotton in a safe and ecofriendly manner, replacing toxic caustic soda used for the purpose earlier (Li and Hardin 1998; Wang et al. 2007; Agrawal et al. 2008a). Lipase in combination with pectinase resulted in the significant reduction of the time required for bioscouring and cotton fabrics with superior properties and excellent dyeing performance were obtained (Kalantzi et al. 2010). Karapinar and Sariisik (2004) reported that, during the bioscouring of cotton with different enzyme combinations, adequate wettability and absorbancy was achieved with cellulase + pectinase and cellulase + pectinase + protease than other enzyme combinations. Klug-Santner and coworkers (2006) reported nearly 80 % removal of pectin from the outer layer of cotton using pectate lyase from *Bacillus pumilus* BK2. While working on bioscouring of cotton fabric with pectinase isolated from *Fusarium* sp., Rajendran and coworkers (2011) found that, the weight of the fabric was reduced up to 0.89 % in comparison with the 4.9 % reduction in weight of the fabric by conventional alkaline scouring. Water absorbing character and tensile strength of the bioscoured fabric was higher than that of conventionally scoured fabric. Vigneswaran and coworkers (2012) reported that, treatment of cotton with alkaline pectinase resulted in water absorbency of <5 s, 52.5 % wax removal and 3.2 % fabric weight loss under optimised conditions (Table 4). Addition of chelating (EDTA) and wetting agent along with pectinase from *Bacillus subtilis* markedly enhanced the weight loss in cotton. Bioscouring of fabric with pectinase resulted in enhancement of various physical properties of fabric viz. whiteness (1.2 %), tensile strength (1.6 %) and tearness (3.0 %) over conventionally alkaline scoured fabrics (Ahlawat et al. 2009). Hartzell and Durrant (2000) studied the effect of agitation on cotton bioscouring by pectinase, and concluded that agitation during scouring improves the fabric wettability. Li and Hardin (1998) studied the effect of surfactants, agitation, and enzyme type, and concluded that, the effect of surfactant and agitation depends on the enzyme structure and the characteristics of cotton fibre. Agrawal and coworkers (2008b) reported the additive effect of *F. solani* pisi cutinase and pectate lyase in cotton bioscouring. Time required for the removal of wax was reduced, when the cutinase (Cutinase 100 U/g of fabric, 30 °C, 50 mM Tris–HCl buffer pH 8, Triton X-100 1 g/L) followed by pectinase (Pectinase 13 U/g of fabric, 50 °C, 50 mM Tris–HCl buffer, pH 8) treatment was used. Study showed that cutinase and pectinase can also be applied effectively together in one reactor vessel.

**Table 4:** Optimised conditions for treatment of various fibres with enzymes

Application	Fibre	Enzymes	pH	Time (h)	Temperature (°C)	Enzyme dose	Moisture content (Fabric to Moisture)	Microorganism	References
Scouring	Cotton	Pectinase	8.5–9	1	60	6 %	–	Commercial	Vigneswaran et al. (2012)
Scouring	Cotton	Pectinase	8	0.33	40	2 %	–	*Fusarium* sp.	Rajendran et al. (2011)
Scouring	Cotton	Lipase, Pectinase	7	0.5	50	50 and 100 U/g	1:40	Commercial	Kalantzi et al. (2010)
Scouring	Cotton, micropoly	Pectinase	9.5	2	65	5U/g	1:20	*Bacillus subtilis*	Ahlawat et al. (2009)
Retting	Hemp, Flax	Pectin lyase	8	24	37	0.24 IU/g	–	*Aspergillus terricola*	Yadav et al. (2009)
Retting	Flax	Pectate lyase	9	1	55	2 %	–	Commercial	Akin et al. (2007)
Degumming	Ramie	Pectate lyase	8.5	2.4	50	40U/g	1:13	*Bacillus* sp.	Guo et al. (2013)
Degumming	Buel	Pectinase	8	24	45	200 U/g	1:5	*Bacillus pumilus* DT7	Kashyap et al. (2001b)
Degumming	Ramie	Pectate lyase, polygalacturonase, xylanase, cellulase	10	5	40	–	–	*Bacillus* sp.	Zheng et al. (2001)
Degumming	Ramie	Pectinase	7	15	–	–	1:40	*Amycolata* sp.	Bruhlmann et al. (1994)

Naturally occurring fibres such as cotton, jute, coir, flax, hemp, ramie and banana are used as raw material for the textile industry (Esfandiari 2007; Kalantzi et al. 2010). The technical feasibility of enzymatic scouring for different fabrics has been recognised by many researchers over the last decade (Tzanov et al. 2001; Lenting et al. 2002; Lu 2005; Moghe and Nabar 2006). The optimised conditions for the treatment of various fibres by pectinolytic enzymes have been represented in Table 4. In addition to pectin, partial removal of the non-fibrous materials like hemicelluloses makes the jute fibre considerably softer. As xylan is a major component of hemicelluloses, so xylanase enzyme is basically used in addition to pectinase. This enzyme allows the selective removal of hemicellulose without affecting the strength of the cellulosic fibre itself. Pectinolytic microorganisms having xylanase activity but devoid of cellulase activity is an additional beneficial aspect to improve the fibre quality of jute (Gomes et al. 1992). Yadav and coworkers (2009) are the first to report the enzymatic retting of hemp and flax using pectin lyase from *Aspergillus terricola* (Table 4). Retting of Latvian hemp sort “Purini” by using pectinase enzyme has been reported by Bernava, (2015). For the manufacture of textiles from ramie fibres, a gum content of <6 % is desired (Bhattacharya and Paul 1976). Decorticated ramie fibres contain about 20–30 % incrusting material consisting mainly of pectin and hemicellulose. Bruhlmann et al. (1994) reported that, the gum content of fresh fibres can be reduced to 15 % when treated with pectinolytic enzyme isolated from *Amycolata* sp. for 15 h. Incubation of ramie fibres with the alkalophilic bacteria, *Bacillus* sp. NT-39, NT-53 and NT-76 resulted in 5.0 % or more loss in the gum content after 48 h, while polysaccharide-degrading enzymes (pectate lyase, polygalacturonase, xylanase and cellulase) from these strains decreased the gum content to 9.4 % after 5 h. Analysis of gum content and enzyme activities revealed that, pectate lyase and xylanase played an important role in the degradation of residual gum. Guo and coworkers (2013) are the first to report the combined effect of enzymatic degumming and H_2_O_2_ bleaching process on ramie fibre. The gum loss and brightness of fibres could be significantly improved, when H_2_O_2_ and pectate lyase from *Bacillus* sp. Y1 were used in combination for degumming.

Treatment of ramie and sunn hemp fibre with combination of polygalacturonase and chemical treatment resulted in release of 9.4 and 7.6 µmol/ml of reducing sugar from ramie and sunn hemp fibre, respectively, whereas reduction in the weight was 37 and 56 %, respectively, after 11 h incubation (Kapoor et al. 2001). Sharma and Satyanarayana (2012) reported that the treatment of ramie fibres with NaOH (0.04 %) followed by the pectinase (300 U/g dry fibre) from *B. pumilus* dcsr1 resulted in the reduction of brittleness, redness, yellowness, and increase in the tensile strength, Young’s Modulus and brightness of the fibre. Combination of chemical and enzymatic process enhances the degumming of bast fibres and decreases the consumption of chemicals and energy (Deshpande and Gurucharanam 1985; Kashyap et al. 2001b). Chemical plus enzymatic process resulted in the release of 575 μmol of galacturonic acid g^−1^ dry fibres after 18 h of treatment (Kashyap et al. 2001b).

### Tea and coffee processing

Pectinase treatment accelerates tea fermentation by breaking down the pectin which is present in the cell walls of tea leaves and also destroys the foam forming property of instant tea powders by destroying the pectins. The change in colour of tea during the fermentation also results in the development of characteristic aroma (Carr 1985; Praveen and Suneetha 2014). Application of cellulase, pectinase and xylanase, isolated from a yeast *Pichia* sp. NRRL Y-4810 and *Zygosaccharomyces* sp. NRRL Y-4882 and the bacterium *Acetobacter* sp. NRRL B-2357, respectively increased the black tea components, theaflavin (TF), thearubigen (TR), caffeine (CAF), high polymerised substances (HPS), total liquor colour (TLC), total soluble solids (TSS) and dry matter content (DMC) over conventional treatment (Murugesan et al. 2002). Marimuthu et al. (1997) reported that use of commercial pectinase and cellulase improves tea quality by increasing 24.77 % TF, 21.52 % TR, 21.54 % HPS and 17.49 % TSS. They have also reported that commercially available pectinase, when used for tea leaf fermentation could increase TF by 5.8 %, TR by 5.72 %, HPS by 4.96 % and TSS by 9.29 % (Marimuthu et al. 2000). Crude enzyme from *Aspergillus* sp. at low concentration (2.5 IU/750 g tea leaf) was more effective in improving the quality of tea than the purified pectinase enzyme at higher concentration (25 IU/750 g tea leaf). This is due to the fact that, the crude enzymes preparation extracted from fungi contains all enzymes, cellulase, hemicellulase, pectinase, proteinase, etc., whereas the purified enzyme preparation contains only pectinase. The crude enzyme from *A. indicus*, *A. falvus* and *A niveus* increase the TF content by 43.81, 62.86 and 59.05 %, respectively, whereas the purified enzyme from these fungi increased the TF content by 38.10, 40 and 34.29 %, respectively. The TLC was enhanced to 18.19, 14.74 and 14.10 % by the crude enzyme from *A. indicus*, *A. falvus* and *A. niveus*, respectively, whereas the purified enzyme from these fungi resulted in an increase of TLC by 12.18, 11.54 and 11.22 %, respectively (Angayarkanni et al. 2002). Senthilkumar and coworkers (2000) reported that, mixed enzyme extract from *A. oryzae*, *A. wentii*, *A. tamari*, *A. japonicus*, *A. awamori* and *Trichoderma koningii* enhanced the tea quality by increasing TF by 45 %, TR by 48 %, HPS by 33 %, TLC by 19 % and TSS by 3 %.

Pectinolytic microorganisms are also used in the fermentation of coffee to remove the mucilaginous coat from the coffee beans. The robusta coffee mucilage layer is gelatinous and viscous in nature, which is surrounded over the bean. It contains 84 % moisture with 8.9 % protein, 4 % sugars, 2.8 % pectin and 0.9 % ash (Murthy and Naidu 2011). The mucilage constitutes about 17 % by mass of the whole cherries. The composition of the robusta coffee pulp could vary depending upon the variety, geographical conditions, management of the estate, etc. (Murthy and Naidu 2011). Degradation of mucilage to sugars contributes to the quality of coffee bean. Pectinases are added to remove the pulpy bean layer consisting of pectic substances. Pectinolysis enabled reduction in demucilisation time which was evident with reduction in pH value and increased sugar release (Murthy and Naidu 2011). Pectinase was produced using coffee pulp and the application of the same was studied on demucilage of coffee pulp, which indicates waste recycle with value addition, that is also economical for coffee industry (Murthy and Naidu 2011). Murthy and Naidu (2011) reported that, crude pectinase from *Aspergillus niger* CFR causes about 54 and 71 % degradation of mucilaginous layer of coffee beans after 1 and 2 h of fermentation process, respectively and complete decomposition of pectin was obtained after 3.5 h.

### Processing of animal feed

The use of pectinases in production of ruminant feed decreases the feed viscosity and increases the absorption of nutrients by ruminants, liberates nutrients by enzymatic action which also reduces the amount of faeces (Hoondal et al. 2002; Praveen and Suneetha 2014). The specific enzyme preparations have become a valuable tool for economically improving the digestive processes in the ruminants (Gado et al. 2009; Murad et al. 2009). Ruminants diet was supplemented by cocktail of enzymes containing xylanases, pectinases and cellulases. Supplementation of enzymes increases the digestibility of organic matter (Selinger et al. 1996; Petersen 2001). Improvement in animal performance due to the use of enzyme additives can be attributed mainly to improvement in ruminal fibre digestion, which results in increased digestible energy intake (Arambel et al. 1987; Ghorai et al. 2009). The net effect of enzyme usage in feed has increased the animal weight. Treatment of feed by spraying enzymes just before feeding provides increased management flexibility. Feed treatment with enzymes in this manner may improve digestibility of the feed via a number of different mechanisms including direct hydrolysis, improvements in palatability, changes in gut viscosity (Ghorai et al. 2009).

### Biobleaching of kraft pulp

With the advancement of biotechnology and increased reliance of paper and pulp industries on the use of enzymes for biobleaching, the use of enzymes like xylanases, ligninases, mannanase and pectinases is increasing in the paper and pulp industries (Kirk and Jefferies 1996; Bajpai 1999). The presence of pectins weakens dewatering during sheet formation due to their high cationic demand and cause yellowness of paper. The pectinases depolymerize polygalacturonic acids and thus decreases the cationic demand in the filtrate from peroxide bleaching of thermo-mechanical pulp (Viikari et al. 2001). Pectinases solely and in combination with other enzymes produced by same or by different microorganisms have been efficiently used for biobleaching of mixed hardwood and bamboo kraft pulps (Ahlawat et al. 2007, 2008; Dhiman et al. 2009; Kaur et al. 2010). The enzyme aided bleaching results in less requirement of bleaching chemicals to attain the same extent of brightness of the pulp as obtained by conventional chemical bleaching and also enhances the physical properties of paper sheet. Reduction in bleaching chemicals would result in reduction of organochlorine compounds in the effluent.

Reduction by 1.2 units in kappa number has been reported by the use of xylanase and pectinase in combination produced from *Bacillus pumilus* and *Bacillus subtilis*, respectively (Ahlawat et al. 2007). Ahlawat et al. (2008) reported the reduction in pulp kappa number and permanganate number by 5.85 and 6.1 %, respectively, after enzymatic treatment of the mixed hardwood and bamboo kraft pulp with pectinase from *Bacillus subtilis* SS. Dhiman et al. (2009) reported 20 % less chlorine consumption after treatment of kraft pulp with xylanase and pectinase in combination produced by different *Bacillus* species. Kaur et al. (2010) also reported that, use of xylano-pectinolytic enzymes extracted from *Bacillus pumilus* resulted in 25 % less chlorine consumption to obtain the same optical properties of the pulp as obtained by conventional chemical bleaching. The synergistic action of xylanase and pectinase which degrades the xylan and pectin present in the pulp fibre and boost up the access of the bleaching chemicals to the lignin present in the pulp by opening up the pulp structure.

### Recycling of wastepaper

Current deinking process depends upon the use of large amount of environment damaging chemicals. Deinking using enzymes is less polluting, energy saving, gives better performance to achieve the desired deinked pulp properties and results in lower disposal problems. Enzymes being used in deinking process are pectinases, hemicellulases, cellulases and lignolytic enzymes. Enzymatic deinking alters bonds near the ink particle and removes the ink from fibre surface. The released ink is then removed by washing or floatation (Xu et al. 2009; Pathak et al. 2010; Xu et al. 2011). A combination of xylanase and pectinase has been used for deinking of school waste paper (Singh et al. 2012). Use of enzymes in deinking results in lower BOD and COD values, thus reduce the cost of waste water treatment in an environment friendly manner (Bajpai and Bajpai 1998; Singh et al. 2012).

## Conclusions

Microbial pectinases are the leading enzyme of the industrial sector. They are being used extensively for various industrial applications and new applications are still coming up. But the main consideration is of enzyme titre and/or stability of the enzyme to make the process cost effective. Production of pectinases has been reported by many workers and cost-effective substrates have also been used but still the production cost is high either due to low activity or instability of enzyme at high temperature for longer duration. So, storage of enzyme at low temperature further increases its cost for industrial application.

The potency of the strain can be increased by enzyme engineering techniques. Enzyme producing companies constantly improve the potency of industrially valuable enzymes producing strains through mutagenesis. Efforts should be made to enhance the activity of enzymes using enzyme engineering approaches. During enzymatic treatment, process is done at particular temperature range and to maintain that range makes the process expensive. Therefore, to reduce the cost of industrial processes, it becomes necessary to use thermostable enzymes. Stability of enzyme over wide range of temperature and pH gives additional advantage to the strain. Further research should be concentrated in increasing the stability of enzyme over wide range of pH and temperature.

So, new microbes with high extracellular pectinase activity, stability over wide range of temperature and pH for a longer period of time, along with their cost-effective production have been the focus of recent research. Immobilisation and reimmobilisation of pectinases onto cost-effective material can have great potential in the clarification of beverages for making the process more cost-effective, so further research should be concentrated in this area also so as to reduce the cost of the enzyme for their efficient use. More research is also needed to discover strains, producing pectinase in combination with other enzymes and the specific combination is required for particular application. This will drastically decrease the production cost for particular application.
